# User-Oriented Requirements for Artificial Intelligence–Based Clinical Decision Support Systems in Sepsis: Protocol for a Multimethod Research Project

**DOI:** 10.2196/62704

**Published:** 2025-01-30

**Authors:** Pascal Raszke, Godwin Denk Giebel, Carina Abels, Jürgen Wasem, Michael Adamzik, Hartmuth Nowak, Lars Palmowski, Philipp Heinz, Silke Mreyen, Nina Timmesfeld, Marianne Tokic, Frank Martin Brunkhorst, Nikola Blase

**Affiliations:** 1 Institute for Health Care Management and Research University of Duisburg-Essen Essen Germany; 2 Department of Anaesthesiology, Intensive Care Medicine and Pain Therapy University Hospital Knappschaftskrankenhaus Ruhr University Bochum Bochum Germany; 3 Knappschaft Kliniken GmbH Recklinghausen Germany; 4 Department of Medical Informatics, Biometry and Epidemiology Ruhr University Bochum Bochum Germany; 5 German Sepsis Society Jena Germany

**Keywords:** medical informatics, artificial intelligence, machine learning, computational intelligence, clinical decision support systems, CDSS, decision support, sepsis, bloodstream infection

## Abstract

**Background:**

Artificial intelligence (AI)–based clinical decision support systems (CDSS) have been developed for several diseases. However, despite the potential to improve the quality of care and thereby positively impact patient-relevant outcomes, the majority of AI-based CDSS have not been adopted in standard care. Possible reasons for this include barriers in the implementation and a nonuser-oriented development approach, resulting in reduced user acceptance.

**Objective:**

This research project has 2 objectives. First, problems and corresponding solutions that hinder or support the development and implementation of AI-based CDSS are identified. Second, the research project aims to increase user acceptance by creating a user-oriented requirement profile, using the example of sepsis.

**Methods:**

The research project is based on a multimethod approach combining (1) a scoping review, (2) focus groups with physicians and professional caregivers, and (3) semistructured interviews with relevant stakeholders. The research modules mentioned provide the basis for the development of a (4) survey, including a discrete choice experiment (DCE) with physicians. A minimum of 6667 physicians with expertise in the clinical picture of sepsis are contacted for this purpose. The survey is followed by the development of a requirement profile for AI-based CDSS and the derivation of policy recommendations for action, which are evaluated in a (5) expert roundtable discussion.

**Results:**

The multimethod research project started in November 2022. It provides an overview of the barriers and corresponding solutions related to the development and implementation of AI-based CDSS. Using sepsis as an example, a user-oriented requirement profile for AI-based CDSS is developed. The scoping review has been concluded and the qualitative modules have been subjected to analysis. The start of the survey, including the DCE, was at the end of July 2024.

**Conclusions:**

The results of the research project represent the first attempt to create a comprehensive user-oriented requirement profile for the development of sepsis-specific AI-based CDSS. In addition, general recommendations are derived, in order to reduce barriers in the development and implementation of AI-based CDSS. The findings of this research project have the potential to facilitate the integration of AI-based CDSS into standard care in the long term.

**International Registered Report Identifier (IRRID):**

DERR1-10.2196/62704

## Introduction

The first clinical decision support systems (CDSS) date back to the 1970s. Early systems, such as MYCIN, a program designed to advise on the choice of therapy selection for patients with infections [1], were rule-based expert systems. Nowadays, a wide variety of CDSS exist. These can be categorized as either knowledge-based or non–knowledge-based systems.

Knowledge-based systems operate on logical decision rules (IF <condition> THEN <action>). The system retrieves data and transforms it into an output following distinct rules. Further segmentation can be made into Bayesian networks, causal-probabilistic networks, and rule-based systems: The latter are usually based on medical guidelines [2].

Non–knowledge-based CDSS require a clinical data source and generate recommendations using artificial intelligence (AI) including machine learning or statistical pattern recognition [3,4]. The potential of AI models to sustainably improve patient care is estimated to be enormous for almost all aspects of the clinical decision-making process (prevention, diagnostics, and therapy) [5]. Based on big data analytics, AI-based CDSS offer the ability to pool, link, and combine data, that would be impossible for humans to interpret due to its complexity. In this way, these models can improve medical outcomes by optimizing care [6].

While AI is established in some disciplines, such as radiology (eg, automated image recognition), the transfer of AI-based CDSS into clinical use is lagging behind. Due to the inhomogeneity of different disease patterns, AI-based CDSS are often developed specifically for a target disease or a selected group of disease patterns, such as sepsis [7,8].

Sepsis is a life-threatening organ dysfunction caused by a dysregulated immune response to infection. It is a leading cause of mortality, with 49 million cases and 11 million deaths each year [9]. So far, only symptomatic therapies are available, that attempt to replace the function of the failed organ systems. Treatment of the dysregulated immune response as a cause of sepsis has not been successful in large trials and subsequently has therefore not found its way into clinical practice or sepsis guidelines.

AI-based CDSS could be particularly useful in sepsis care due to the high heterogeneity and complexity of the disease [10]. Non–knowledge-based respectively data-based CDSS are subject to a trade-off between model complexity and interpretability. As sepsis is an extremely complex condition, a majority of machine learning–based CDSS for this disease can be considered “black box” systems. Their treatment recommendations cannot or can only be interpreted by health care providers, with relatively high effort [11,12]. Health care providers may have to rely on these systems without understanding how the algorithms reach their conclusions, due to their black box nature. This lack of transparency can negatively impact the acceptability of such systems [13-15].

In addition to the black box nature of AI-based CDSS, there may be other possible reasons why such systems do not manage the transition into standard care, such as a nonuser-oriented development approach without or at least without sufficient consideration of the needs and preferences of future users [16-18], resulting in reduced user acceptance and implementation barriers (eg, computer literacy of the future users, data availability or legal issues) [4].

These may be possible reasons why there is still no AI-based CDSS for sepsis in Germany that is included in the standard care of the statutory health insurance (SHI) system and is used nationwide. Currently, only a few prototypes in the form of individual solutions are in use or under development (eg, [19,20]).

The reluctance to adopt AI-based CDSS does not appear to stem from the performance of such systems. The sepsis prediction algorithm InSight, developed by AI start-up Dascena, has been demonstrated in several articles to outperform traditional rule-based scores such as the Systemic Inflammatory Response Syndrome, the Sequential Organ Failure Assessment or the quick Sequential Organ Failure Assessment [21,22]. This and other developed algorithms have the potential to improve patient-relevant outcomes. In particular, this encompasses a reduction of sepsis-related mortality, a reduced average length of hospitalization, or an earlier treatment, for example, in the form of the timely administration of antibiotics [21,23,24]. Instead, the reluctance to implement AI-based CDSS can be attributed to a number of factors that are independent of their performance. These include a paucity of evidence, particularly prospective studies [22], a lack of capacity in health care systems to integrate AI into current workflows [25], and ethical concerns, such as the risk of discrimination against certain populations [25-27]. Also in other indications, despite a high frequency of development, only a marginal proportion of such systems successfully transition from the development phase into standard care.

Therefore, the multimethod research project “User-Oriented Requirement Profile for AI-Based Clinical Decision Support Systems Using the Medical Example of Sepsis – KI@work,” seeks to investigate AI-based CDSS in the above-mentioned disease context. In the framework of this research project, it is assumed that there are 2 reasons for the lack of implementation. First, there are administrative and organizational barriers (data availability, data collection, knowledge gaps among potential users) as well as legal and institutional hurdles (implementation of European and national legal requirements, [medical] liability law, competent bodies) within the German health care system that make it difficult to transfer and integrate AI-based CDSS into the SHI system. Second, to ensure (sustainable) use and acceptance of AI-based CDSS, the system must have a high perceived usefulness according to the Technology Acceptance Model [28]. In addition, future users should be involved in the development phase, as suggested by the Recursive Innovation Management Model [18]. Due to the strongly technology-driven development of AI-based CDSS, this is currently only done in a fragmentary manner, so that the requirements and preferences of users are only insufficiently taken into account within the framework of such systems.

The multimethod research project addresses both aspects, resulting in two equally important research objectives, that are (1) to identify and remove or overcome barriers by developing health policy recommendations for action to facilitate the transfer of AI-based CDSS across all indications in the German health care system in the future and (2) to develop a clinical requirement profile that can be incorporated into the initial development of CDSS or can be considered in the further development of existing systems. This should enable an increase in usability and thus the acceptance of AI-based CDSS. The requirement profile is developed using the example of sepsis and is, therefore, indication-specific.

In order to achieve the objectives, 3 research questions and 3 sub-questions were determined (Textbox 1).

**Research questions of the multimethod research project.**

- What insights can be gained from AI-based clinical decision support systems (CDSS) that are already established in health care and which best practices can be derived?
  What is the data basis of these CDSS (input)?
  How are the decisions and recommendations of the CDSS presented to the health care providers (output)?
  How does the interaction between health care providers and CDSS take place (setting)?
- What specific problems exist or are seen in the establishment of AI-based CDSS in patient care, with a particular focus on clinical sepsis care as well as on the German health care system?
- What are the preferences of health care providers regarding the use and design of CDSS in the prevention, diagnosis, and treatment of patients with sepsis?

The research project is conducted by the Institute for Health Care Management and Research at the University of Duisburg-Essen. Consortium partners are the Department of Anesthesiology, Intensive Care Medicine and Pain Therapy at the University Hospital Knappschaftskrankenhaus Bochum, the Knappschaft Kliniken GmbH, the Department of Medical Informatics, Biometry and Epidemiology at the Ruhr University Bochum and the German Sepsis Society. The research project is funded by the Innovation Fund of the German Joint National Committee (funding code: 01VSF22050).

## Methods

### Overview

**Figure 1:**
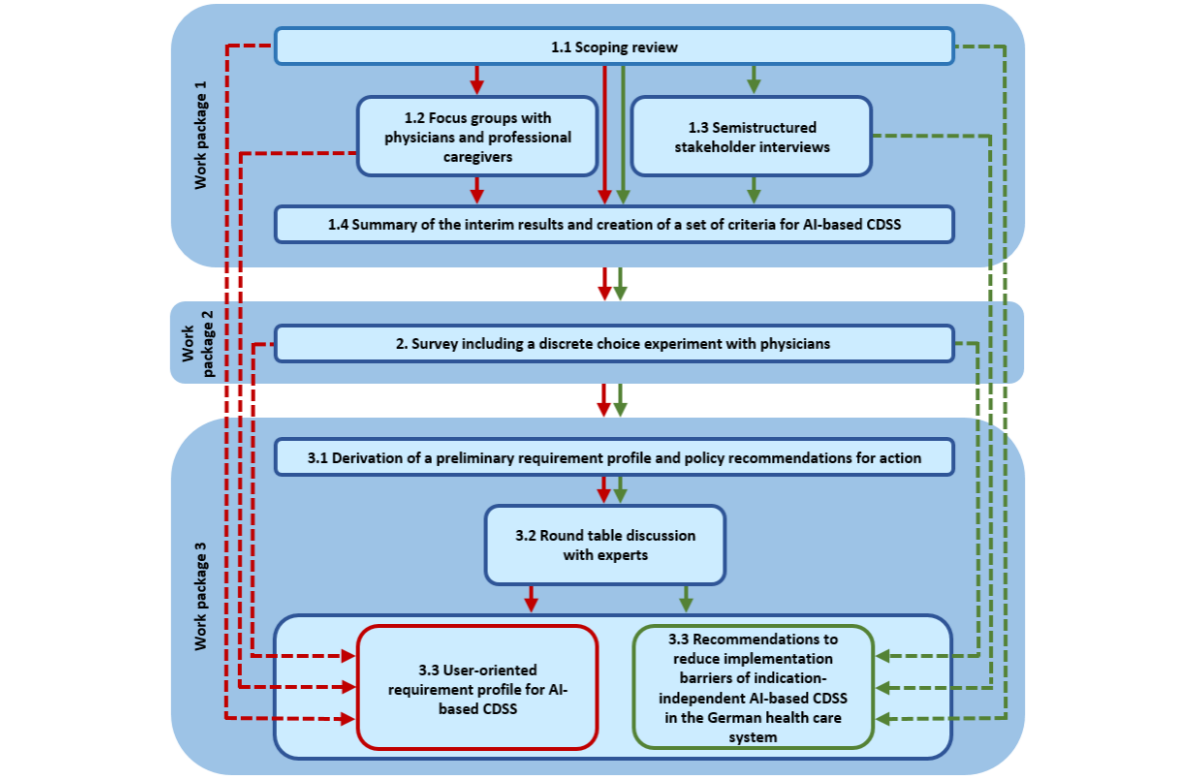
Overview of the multimethod research project. The results of work packages 1 and 2 are indirectly incorporated into module 3.3, as indicated by the dashed lines. AI: artificial intelligence; CDSS: clinical decision support system.

The research project is conducted over a period of 36 months (cf Multimedia Appendix 1) and uses a multimethod approach. It is separated into 3 work packages. Work package 1 combines a scoping review, focus groups with physicians and professional caregivers, and semistructured interviews with relevant stakeholders of the German health care system. At the end of this work package, the interim results (problems, barriers, and corresponding solutions) of the research project are summarized and a set of criteria for AI-based CDSS is derived. Based on the results of the preceding work package, work package 2 includes the central element of the research project: a survey of physicians, including a discrete choice experiment (DCE). Work package 3 involves the development of a requirement profile for AI-based CDSS and the derivation of health policy recommendations for action, which are discussed in an expert roundtable discussion. The research project concludes with a summary of the results in a white paper (cf Figure 1).

### Work Package 1

Work package 1 addresses both research objectives. It serves to identify problems and barriers regarding the transfer of AI-based CDSS into the SHI system (first research objective) and to create a preliminary set of criteria for AI-based CDSS in sepsis care (second research objective). Work package 1 is divided into 4 modules.

#### Scoping Review (Module 1.1)

The scoping review combines systematic and structured research. The focus of the scoping review lies on research questions 1 and 2, thus addressing both research objectives.

##### Scoping Review

The actual scoping review examines the currently available evidence on the patient-relevant benefit of AI-based CDSS in the field of sepsis. Furthermore, it aims to identify factors that pose barriers to the transition of CDSS into the health care system and to identify solutions that reduce or overcome these barriers. Methodically it is based on the Joanna Briggs Manual for Evidence Synthesis [29]. Further development of the foundational work of Arksey and O'Malley [30] and Levac et al [31]. The documentation of the scoping review is based on the PRISMA Extension for Scoping Reviews [32]. The search strategy is designed using the PCC (Population, Concept, and Context) framework. The population encompasses individuals with or at risk of sepsis, the concept used is AI and the context are CDSS. Relevant search terms according to the predefined PCC framework are identified and linked with “OR” operators in order to form search blocks. The search blocks are linked with “AND” operators to generate search strings. All search terms are restricted to their occurrence in the abstract, the title, or as a keyword, and in the case of existing index terms (eg MeSH and Emtree), the corresponding terms are added. The databases examined are Medline and Embase as well as ACM Digital Library and IEEE Xplore. This proceeding is to ensure that the interdisciplinary character of the research project can be adequately investigated from both the medical and the informatics perspectives. Following the identification and removal of duplicates, 2 reviewers (GDG and PR) independently screen the titles and abstracts against predefined inclusion and exclusion criteria (cf Textbox 2) to determine whether an article is eligible for full-text screening. Subsequently, the same 2 reviewers conduct a full-text screening of the included articles against the same criteria.

The reviewer PR then uses the program MAXQDA (VERBI Software GmbH) to identify and tag relevant content in the included articles. The final categories are then discussed and systematized in a workshop (NB, GDG, and PR). The results of the workshop are recorded in Microsoft Excel.

In order to add further evidence, the reference lists of the articles identified in the scientific databases are screened. In addition, a structured search is conducted for gray literature (eg, working papers and guidelines) from various governmental and nongovernmental stakeholders, such as associations or public institutions, and their websites.

**Inclusion and exclusion criteria for the scoping review.**

Inclusion criteria

- Articles focusing on sepsis and
- involving AI-based clinical decision support systems, that
  describe patient-related benefits
  describe problems with development, implementation, or application, or
  describe approaches to overcome identified problems

Exclusion criteria

- Exclusively technical description of systems.
- Focus on the description of the evaluation of binary classifiers.
- Articles describing AI-based clinical decision support systems for neonates and children or animals.
- Not addressing any of the research questions in more detail.
- Research protocols, conference abstracts, letters to the editor, or expression of opinions.
- Article published before 2008.
- Language other than English or German.

##### Additional Structured Medical Device Database Search

The additional structured search aims to provide an overview of authorized AI-based CDSS already in use (irrespective of the indication area of sepsis). In order to identify such systems, the European Database on Medical Devices is analyzed. The filters “system,” “software,” and “risk class (IIa, IIb, III)” are used. The structured search examines the data on which the decisions of the systems are based (input), how the results are presented (output), and how the CDSS are integrated into the clinical context (setting). Particular focus is placed on the search for best practice examples and the identification of clinical areas where the use of AI is already established. These examples are used to recognize aspects that increase the likelihood of such systems being implemented.

#### Focus Groups With Physicians and Professional Caregivers (Module 1.2)

The focus groups with physicians and professional caregivers build on the interim findings of the scoping review (module 1.1). The findings of the focus groups contribute to the preparation of a standardized questionnaire for the survey in work package 2. Relevant aspects of input, output, and setting in the context of AI-based CDSS in the field of sepsis diagnosis and therapy are collected and derived, thus addressing the second research objective.

Five web-based focus groups are held using the conferencing tool integrated in Microsoft Teams. The participants include physicians and professional caregivers familiar with the care and treatment of adult patients with sepsis, as well as those who contribute to the prevention and diagnosis of sepsis. During the process of participant recruitment, attention is paid to ensure the inclusion of a heterogeneous group of hospitals, representing various levels of care (from primary to quaternary care). In addition, a balanced composition of focus groups in terms of gender is prioritized. Furthermore, participants with differing levels of experience are integrated into the individual focus groups. Three focus groups are conducted with physicians and 2 with professional caregivers. The discussions are based on semistructured guidelines. The focus groups are led by a moderator team according to Krueger and Casey [33], recorded and transcribed. They are then subjected to thematic qualitative content analysis based on Kuckartz and Rädiker [34] using the program MAXQDA (VERBI Software GmbH). The aspects of input, output, and setting represent deductive codes and are defined before data analysis. Inductive codes are supplemented during the process of analysis. A final categorization is conducted in a workshop (NB, GDG, and PR). The findings of the workshop are recorded in Microsoft Excel.

Recruitment of participants is supported by the Department of Anesthesiology, Intensive Care Medicine, and Pain Therapy of the University Hospital Knappschaftskrankenhaus Bochum and the Knappschaft Kliniken GmbH. In addition, participants from other hospital providers are also invited in order to represent a broad and provider-independent perspective of physicians and professional caregivers.

#### Semistructured Stakeholder Interviews (Module 1.3)

Semistructured interviews with stakeholders from different domains of the German health care system are conducted to complement the results of the scoping review (module 1.1) with the perspectives of various stakeholders. The expert interviews are undertaken to identify problems and barriers related to the implementation of AI-based CDSS in standard care (first research objective). Experts in the field of medical device law, representatives of the SHI system, patient representatives, physicians, and professional caregivers, representatives of quality management, data protection, and ethics, and various research institutions as well as private developers are interviewed. Similar to the focus groups, the interviews are recorded, transcribed, and subjected to qualitative content analysis according to Kuckartz and Rädiker [34].

#### Summary of the Interim Results (Problems, Barriers, and Corresponding Solutions) and Creation of a Set of Criteria for AI-Based CDSS (Module 1.4)

Based on the results of the first work package, a set of criteria for AI-based CDSS in sepsis care is developed in module 1.4 (second research objective), which is based on national and international evidence and qualitative survey methods. Furthermore, the identified problems and barriers as well as corresponding solutions are systematized and summarized (first research objective).

### Work Package 2

The central element of work package 2 is a cross-sectional survey to identify perceived problems and barriers to the integration and usage of AI-based CDSS (first research objective) and to ascertain physicians’ preferences regarding the design of AI-based CDSS in sepsis care (second research objective). The results of work package 1 serve as a basis for the development of the survey, including the DCE.

The questionnaire is divided into 3 sections. First, sociodemographic data and general attitudes toward AI applications are collected; the second section aims to identify potential barriers to the integration of AI-based CDSS into care; and third, a DCE is conducted to determine preferences for the criteria in the preliminary AI requirement profile. In particular, preferences are sought regarding the preferred information content and the appropriate integration into the care process.

The sociodemographic section comprises personal details, such as age groups or gender of the respondents, in addition to occupational data. This includes, for instance, professional experience in intensive care medicine (in 5 groups from none to >10 years), the medical specialty in which the participants are active as well as an evaluation of the degree of digitalization within the hospital where the respondents work. This procedure ensures the anonymity of the participants. The section concludes with an evaluation of general attitudes toward AI.

The second section of the questionnaire focuses on the potential barriers and problems associated with the integration of AI-based CDSS into standard care. It begins with questions concerning non–AI-based CDSS and then progresses to questions related to the use of AI in health care in general. The section concludes with questions on AI-based CDSS. In this part of the questionnaire, problems are primarily assessed using the Likert scale.

The last section of the questionnaire is intended to ascertain the requirements and preferences of physicians with regard to AI-based CDSS. For this purpose, a DCE is conducted to determine preferences regarding the design of such systems. Participants are presented with 2 fictitious systems and asked to indicate their preference.

The sample size of the survey could not be defined before the development of the questionnaire as it depends on the attributes queried in the DCE in the third section. The attributes are derived from the results of work package 1 and were thus not available at the time of the preparation of the grand proposal. Moreover, no comparable research projects could be identified from which recommendations for the required sample size and estimates of the utility of the items could have been derived [35]. Nevertheless, for orientation, initial sample size planning was done according to the heuristics developed by Johnson and Orme [36]. Therefore, 12 choice decisions, 2 selection sets per task, and a maximum of 3 levels were assumed, for which 125 evaluable questionnaires must be available. Since an additional evaluation according to subgroups such as gender (male, female, or diverse), age, or occupational group is to be conducted, the necessary number increases to 125×8=1000 completed questionnaires. Once the exact number of choice decisions, tasks, and levels is known, a statistical assessment is made to ensure statistical power for the given sample size.

In accordance with Pöge et al [37] gender identity is used as a binary variable, so transgender and cisgender people are evaluated together. Gender-diverse people are not reported separately in order to avoid identifiability due to the expected low number of cases but are included in the overall category of all respondents.

A total of 6667 physicians must be contacted, assuming an average response rate of 15% (1000/6667). Therefore, approximately 400 members of the German Sepsis Society and 250 physicians from the Knappschaft Kliniken GmbH who fulfill the inclusion criteria—(1) familiarity with adult medical care and (2) experience in intensive care medicine—are included. The sample is supplemented by randomly selected records of an address register. Based on the inclusion criteria, only addresses of physicians from the medical specialties of intensive care medicine, anesthesiology, orthopedics and trauma surgery, general surgery, visceral surgery, and internal medicine are selected. The Knappschaft Kliniken GmbH hospitals are excluded during the address data extraction process of the address register, as the hospital addresses are known and can be defined as an exclusion criterion to prevent duplicates. Furthermore, a duplicate check is conducted between the address register and the German Sepsis Society to avoid duplicates and to ensure that all physicians are not contacted twice.

In order to reach “offliners,” it is possible to take part in the survey both online (LimeSurvey) and on paper. A pretest is conducted before the distribution of the survey in order to identify potential deficiencies and implement necessary modifications. A think-aloud protocol is used to ascertain the comprehensibility, manageability, completeness, and time required to complete the questionnaire [38]. In addition, the online version undergoes several functional tests for layout and usability.

Univariate or bivariate descriptive analyses are carried out as part of the evaluation of the second part of the questionnaire. Depending on the type of variable, frequency distributions, mean values, or medians are compared with simultaneous testing of statistical significance. Subgroup analyses are also conducted, for example, in relation to age or gender. The DCE (third section of the questionnaire) is expected to be analyzed using logit or mixed logit models. As both the absolute influence of the attributes and the relative influence of the levels are of interest, dummy coding is used.

### Work Package 3

In work package 3, a white paper is developed. It includes (1) the final requirement profile for AI-based CDSS and (2) the determined health policy recommendations for action to reduce implementation barriers. Therefore, a preliminary requirement profile and health policy recommendations for action are developed (module 3.1), discussed with experts (module 3.2), and finally summarized in a white paper (module 3.3).

#### Derivation of a Preliminary Requirement Profile and Health Policy Recommendations for Action (Module 3.1)

The preliminary requirement profile for AI-based CDSS in the treatment of sepsis is developed based on the results of module 1.4 (summary of interim results and creation of a set of criteria for AI-based CDSS) as well as the results of the survey from work package 2 (second research objective). Furthermore, the identified barriers to the implementation and integration of AI-based CDSS in the German SHI system are used to develop targeted strategies for the removal and reduction of implementation barriers and translated into health policy recommendations for action (first research objective).

#### Expert Roundtable Discussion on the Requirement Profile and Corresponding Health Policy Recommendations for Action (Module 3.2)

In module 3.2 an expert roundtable is held. The aim of the discussion is to evaluate and optimize the preliminary requirement profile for AI-based CDSS as well as the corresponding health policy recommendations for action. In order to gain a comprehensive perspective, different stakeholders involved in the development and provision of AI-based CDSS are invited. In the context of the requirement profile for AI-based CDSS for sepsis care, the expert roundtable discussion focuses on input (data basis), output (presentation of decisions or recommendations), and setting (context of interaction between AI-based CDSS and user). Besides technical requirements, the results can include further requirements such as organizational, procedural, legal, or medical content.

The discussion is divided into four parts, after (1) introductory presentations, the (2) preliminary requirement profile for AI-based CDSS and (3) the health policy recommendations for action for the use of these systems in the German SHI system are presented. The workshop then provides an opportunity for (4) open discussion of the partial results of the requirement profile and the health policy recommendations. In addition, selected topics can be discussed in small groups with relevant experts, and the results of the discussions are presented in a plenum to reach a consensus among the stakeholders on the main issues.

A maximum of 30 stakeholders are invited to the workshop. In addition, there are at least 2 moderators and a technical and organizational staff member from the Institute for Health Care Management and Research from the University of Duisburg-Essen, as well as representatives from the University Hospital Knappschaftskrankenhaus Bochum and the German Sepsis Society. Discussions are led by a team of facilitators using prepared guidelines. The results of the workshop are recorded, transcribed, and subsequently analyzed.

#### Finalization of the Requirement Profile as Well as the Health Policy Recommendations for Action and Preparation of a White Paper (Module 3.3)

Based on the results of the expert roundtable discussion (module 3.2), the requirement profile for AI-based CDSS is finalized using the example of sepsis care. This enables a user-oriented development of AI-based CDSS in this context. Wherever possible, generic aspects are elaborated and presented in order to include indication-independent and therefore generalizable information in the requirement profile.

In addition, the health policy recommendations for action are concretized. It is discussed how to reduce or overcome barriers to the implementation and establishment of AI-based CDSS in clinical care and finally, proposals for legal adaptations are derived.

The results of both research project objectives, the requirement profile for an AI-based CDSS, and the health policy recommendations for action, are published in a white paper.

### Ethical Considerations

In agreement with the ethical review committee of the Medical Faculty of the University of Duisburg-Essen, an ethics vote is not required as only physicians and experts are surveyed or interviewed within the project and no patient data is collected or used.

## Results

The research project started in November 2022. The scoping review has been completed and the qualitative modules have been subjected to analysis.

As part of the scoping review, factors that pose barriers to the transition of CDSS into the health care system as well as solutions that reduce or overcome these barriers are analyzed. The review also sought to investigate the potential patient-relevant benefits of AI-based CDSS. The scoping review thus serves both to develop the guidelines for the qualitative modules and to derive potential problems for the survey (section 2 of the questionnaire).

The expert interviews, conducted between June and August 2023, aim to identify further problems and possible solutions for AI-based CDSS in the context of the German SHI system. The findings derived from the expert interviews are subsequently employed in the development of the survey (section 2 of the questionnaire).

The objective of the focus groups, conducted in June 2023, is to ascertain the preferences and requirements of health care providers with regard to the input, output, and setting of AI-based CDSS. The findings of the focus groups are used in the development of the DCE (section 3 of the questionnaire).

The conception of the survey, including the DCE, is finalized. It focuses on the preferences and requirements of physicians regarding the design of AI-based CDSS and the potential problems associated with their implementation. The questionnaire is subjected to a series of comprehensive pretests. Furthermore, it is implemented on the web-based survey platform LimeSurvey (LimeSurvey GmbH) to facilitate digital participation.

Recruitment of the 6667 survey participants was initiated at the end of July 2024 and the results of the scoping review, and the qualitative modules are expected to be published at the end of 2024.

## Discussion

### Principal Findings

AI-based CDSS are developed for various diseases. These systems possess the potential to enhance the quality of care and thereby positively impact patient-relevant outcomes (such as a reduction of sepsis-related mortality, a reduced average hospital length of stay, or an earlier administration of antibiotics) [21,23,24]. Nonetheless, despite extensive development efforts, the majority of CDSS developed have not been adopted in standard care and do not make a significant contribution to improving care in their current form.

There may be 2 primary reasons for this. First, it is assumed that a technology push development is currently taking place, wherein AI-based CDSS are being developed without or only insufficiently considering the requirements and preferences of users [16]. Such requirements may relate to the complexity of the algorithm, with simpler algorithms potentially being preferred to more complex approaches that are incomprehensible and may be perceived as black boxes by users. Requirements related to the design and layout of AI-based CDSS may vary depending on professional experience or age. An analysis of current evidence reveals that other authors have identified various requirements for AI-based CDSS among health care providers in qualitative studies. These criteria include ease of implementation, predictive capability, and costs [39]. In addition, workload requirements [40] and the need for training programs [41] have also been highlighted.

Second, the implementation of such systems may face various barriers during the transition from the developmental phase to standard care, for instance, operational problems or regulatory uncertainties [4]. These and other hindrances may have a negative effect on the successful integration of AI-based CDSS and need to be identified and addressed before AI-based CDSS can be sustainably integrated into care.

Based on these 2 hypotheses it is necessary to analyze potential barriers, as well as the requirements and preferences of health care providers for AI-based CDSS. The results are summarized in health policy recommendations for action to reduce barriers and a requirement profile for AI-based CDSS in order to develop user-oriented systems and thereby optimize user acceptance.

Since the requirements for AI-based CDSS vary depending on the indication, the requirement profile is developed using the specific example of sepsis. Sepsis is a suitable subject for investigation due to its heterogeneity and complex pathophysiological processes, which pose challenges for health care providers in terms of diagnosis and treatment [10]. In addition, the intensive medical treatment and the continuous monitoring of patients with sepsis in the intensive care unit generate a large amount of data suitable for use in AI-based analysis.

The requirement profile for sepsis-specific AI-based CDSS, which is developed based on the requirements and preferences of health care providers, can help to ensure that future CDSS development is aligned with medical practice needs. Involving future users of such systems may counteract the current technology-push development and contribute to greater user acceptance. Following completion, the requirement profile is evaluated in terms of generalizability, and transferability to other indications. In addition, indication-independent health policy recommendations for action are developed based on the identified inhibiting factors for the implementation of AI-based CDSS.

### Strengths and Limitations

The results of this multimethod research project, combining qualitative and quantitative research methods, represent the first attempt to create a comprehensive user-oriented requirement profile for the development of sepsis-specific AI-based CDSS. In addition, general recommendations are derived to reduce barriers to the development and implementation of such systems. Thus, this research project has the potential to promote future technology and facilitate the transfer of AI-based CDSS into standard care.

Despite the comprehensive design of the research project, it is not free of limitations. International literature is analyzed in the scoping review to provide an international perspective. However, all subsequent modules are limited to the perspective of health care providers and stakeholders in the German health care system, which may limit international comparability.

Qualitative and quantitative methods each have inherent limitations. Combining both is expected to leverage their strengths and mitigate their weaknesses. For instance, the predominantly qualitative findings from work package 1, which tend to be subjective and nongeneralizable, are tested for generalizability through the quantitative survey conducted in work package 2. However, not all nuances obtained through the qualitative approaches can be fully reflected in the quantitative survey. The combination of various research approaches—scoping review, expert interviews, focus groups, quantitative survey, and expert workshop—ensures that the project delivers comprehensive and well-founded results.

Furthermore, recruitment for the survey and the DCE may lead to selection bias. Although the majority of respondents are randomly selected from an address register, there is a risk of overrepresentation of physicians from the German Sepsis Society and Knappschaft Kliniken GmbH.

### Dissemination Plan

The dissemination plan for this research project includes publishing the findings of the scoping review, the qualitative modules, the survey including the DCE, and the white paper in peer-reviewed open access journals dedicated to health informatics, digital health, data science, and emerging health technologies. The results will also be presented at national and international conferences focusing on digitalization, sepsis, and health services research.

### Conclusion

Based on the results of this research project, developers are provided with guidelines for the development of new AI-based CDSS or the revision of existing systems in order to make their products more user-oriented. In addition, the research project culminates in the development of health policy recommendations for action to reduce barriers to the implementation of AI-based CDSS. Ultimately, this enables AI-based CDSS to become a future standard in health care practice, providing benefits to patients.

## Appendix

Multimedia Appendix 1 Timeline of the multimethod research project.

## Abbreviations

AI: artificial intelligence
CDSS: clinical decision support system
DCE: discrete choice experiment
PCC: Population, Concept, and Context
SHI: statutory health insurance

## Data Availability

The datasets generated during and/or analyzed during this study are not publicly available for privacy reasons but are available from the corresponding author on reasonable request.

## References

[1] Shortliffe EH, Buchanan BG (1975). A model of inexact reasoning in medicine. Mathematical Biosciences.

[2] Börm P (2021). Leitlinienbasierter Clinical Decision Support – Anforderungen an evidenzbasierte Entscheidungsunterstützungssysteme. OP-JOURNAL.

[3] Sheikhtaheri A, Sadoughi F, Hashemi Dehaghi Z (2014). Developing and using expert systems and neural networks in medicine: a review on benefits and challenges. J Med Syst.

[4] Sutton RT, Pincock D, Baumgart DC, Sadowski DC, Fedorak RN, Kroeker KI (2020). An overview of clinical decision support systems: benefits, risks, and strategies for success. NPJ Digit Med.

[5] Giordano C, Brennan M, Mohamed B, Rashidi P, Modave F, Tighe P (2021). Accessing artificial intelligence for clinical decision-making. Front Digit Health.

[6] Bezemer T, de Groot MC, Blasse E, Ten Berg MJ, Kappen TH, Bredenoord AL, van Solinge WW, Hoefer IE, Haitjema S (2019). A human(e) factor in clinical decision support systems. J Med Internet Res.

[7] Berner E, La Lande LT, Berner ES (2007). Overview of clinical decision support systems.

[8] Peiffer-Smadja N, Rawson TM, Ahmad R, Buchard A, Georgiou P, Lescure FX, Birgand G, Holmes AH (2020). Machine learning for clinical decision support in infectious diseases: a narrative review of current applications. Clin Microbiol Infect.

[9] World Health Organization (2020). Global report on the epidemiology and burden of sepsis. Current evidence, identifying gaps and future directions.

[10] Singer M, Deutschman CS, Seymour CW, Shankar-Hari M, Annane D, Bauer M, Bellomo R, Bernard GR, Chiche J, Coopersmith CM, Hotchkiss RS, Levy MM, Marshall JC, Martin GS, Opal SM, Rubenfeld GD, van der Poll Tom, Vincent J, Angus DC (2016). The third international consensus definitions for sepsis and septic shock (Sepsis-3). JAMA.

[11] Garnica O, Ruiz-Giardín J, Hidalgo J, Boyko N, Golubnitschaja O (2023). Artificial intelligence-based predicitve, preventive, and personalised medicine applied to bacteraemia diagnosis. Microbiome in 3P Medicine Strategies. The First Exploitation Guide.

[12] Kuhn M, Johnson K (2013). Applied predictive modeling.

[13] Durán JM, Jongsma KR (2021). Who is afraid of black box algorithms? On the epistemological and ethical basis of trust in medical AI. J Med Ethics.

[14] Petersson L, Larsson I, Nygren JM, Nilsen P, Neher M, Reed JE, Tyskbo D, Svedberg P (2022). Challenges to implementing artificial intelligence in healthcare: a qualitative interview study with healthcare leaders in Sweden. BMC Health Serv Res.

[15] Khairat S, Marc D, Crosby W, Al Sanousi Ali (2018). Reasons for physicians not adopting clinical decision support systems: critical analysis. JMIR Med Inform.

[16] Mockenhaupt A (2021). Digitalisierung und Künstliche Intelligenz in der Produktion. Grundlagen und Anwendung.

[17] Carroll C, Marsden P, Soden P, Naylor E, New J, Dornan T (2002). Involving users in the design and usability evaluation of a clinical decision support system. Comput Methods Programs Biomed.

[18] Frank K, Reitmeister P (2003). Rekursives Innovationsmanagement.

[19] (2019). Qualitätssicherungsverfahren. Diagnostik, Therapie und Nachsorge der Sepsis. Institut für Qualitätssicherung und Transparenz im Gesundheitswesen (IQTIG).

[20] Kinbiotics.

[21] Shimabukuro DW, Barton CW, Feldman MD, Mataraso SJ, Das R (2017). Effect of a machine learning-based severe sepsis prediction algorithm on patient survival and hospital length of stay: a randomised clinical trial. BMJ Open Respir Res.

[22] Burdick H, Pino E, Gabel-Comeau D, McCoy A, Gu C, Roberts J, Le S, Slote J, Pellegrini E, Green-Saxena A, Hoffman J, Das R (2020). Effect of a sepsis prediction algorithm on patient mortality, length of stay and readmission: a prospective multicentre clinical outcomes evaluation of real-world patient data from US hospitals. BMJ Health Care Inform.

[23] Adams R, Henry KE, Sridharan A, Soleimani H, Zhan A, Rawat N, Johnson L, Hager DN, Cosgrove SE, Markowski A, Klein EY, Chen ES, Saheed MO, Henley M, Miranda S, Houston K, Linton RC, Ahluwalia AR, Wu AW, Saria S (2022). Prospective, multi-site study of patient outcomes after implementation of the TREWS machine learning-based early warning system for sepsis. Nat Med.

[24] Hsu K, Cho D, Hsueh P, Yu J, Sun P (2023). Development of a comprehensive intelligent antimicrobial system: an epochal, fast, and digitally precise prediction of therapeutic antibiotics. Case Study.

[25] Wu M, Du X, Gu R, Wei J (2021). Artificial intelligence for clinical decision support in sepsis. Front Med (Lausanne).

[26] Wang H, Li Y, Naidech A, Luo Y (2022). Comparison between machine learning methods for mortality prediction for sepsis patients with different social determinants. BMC Med Inform Decis Mak.

[27] Zentrale E (2021). Zentrale Ethikkommission Deutsches Aerzteblatt. Dtsch Aerztebl.

[28] Davis FD, Bagozzi RP, Warshaw PR (1989). User acceptance of computer technology: a comparison of two theoretical models. Management Science.

[29] Aromataris E, Lockwood C, Porritt K, Pilla B, Jordan Z (2024). JBI Manual for Evidence Synthesis.

[30] Arksey H, O'Malley L (2005). Scoping studies: towards a methodological framework. International Journal of Social Research Methodology.

[31] Levac D, Colquhoun H, O'Brien KK (2010). Scoping studies: advancing the methodology. Implement Sci.

[32] Tricco AC, Lillie E, Zarin W, O'Brien KK, Colquhoun H, Levac D, Moher D, Peters MD, Horsley T, Weeks L, Hempel S, Akl EA, Chang C, McGowan J, Stewart L, Hartling L, Aldcroft A, Wilson MG, Garritty C, Lewin S, Godfrey CM, Macdonald MT, Langlois EV, Soares-Weiser K, Moriarty J, Clifford T, Tunçalp Ö, Straus SE (2018). PRISMA extension for scoping reviews (PRISMA-ScR): checklist and explanation. Ann Intern Med.

[33] Krueger RA, Casey MA (2014). Focus groups. A Practical Guide for Applied Research.

[34] Kuckartz U, Rädiker S (2022). Qualitative Inhaltsanalyse. Methoden, Praxis, Computerunterstützung.

[35] Assele SY, Meulders M, Vandebroek M (2023). Sample size selection for discrete choice experiments using design features. Journal of Choice Modelling.

[36] Johnson R, Orme B (2003). Sawtooth Software Inc. Getting the most from CBC.

[37] Pöge K, Rommel A, Starker A, Prütz F, Tolksdorf K, Öztürk I, Strasser S, Born S, Saß A (2022). Erhebung geschlechtlicher Diversität in der Studie GEDA 2019/2020-EHIS – Ziele, Vorgehen und Erfahrungen. J Health Monit.

[38] Häder M (2006). Empirische Sozialforschung: Eine Einführung.

[39] Joshi M, Mecklai K, Rozenblum R, Samal L (2022). Implementation approaches and barriers for rule-based and machine learning-based sepsis risk prediction tools: a qualitative study. JAMIA Open.

[40] Manetti S, Cumetti M, De Benedictis A, Lettieri E (2022). Adoption of novel biomarker test parameters with machine learning-based algorithms for the early detection of sepsis in hospital practice. J Nurs Manag.

[41] Sandhu S, Lin AL, Brajer N, Sperling J, Ratliff W, Bedoya AD, Balu S, O'Brien C, Sendak MP (2020). Integrating a machine learning system into clinical workflows: qualitative study. J Med Internet Res.

