# Supplementary figures and images for: User-Oriented Requirements for Artificial Intelligence–Based Clinical Decision Support Systems in Sepsis: Protocol for a Multimethod Research Project
Source: JMIR Res Protoc. 2025 Jan 30;14:e62704. doi: 10.2196/62704 (PMC11826947; doi:10.2196/62704)

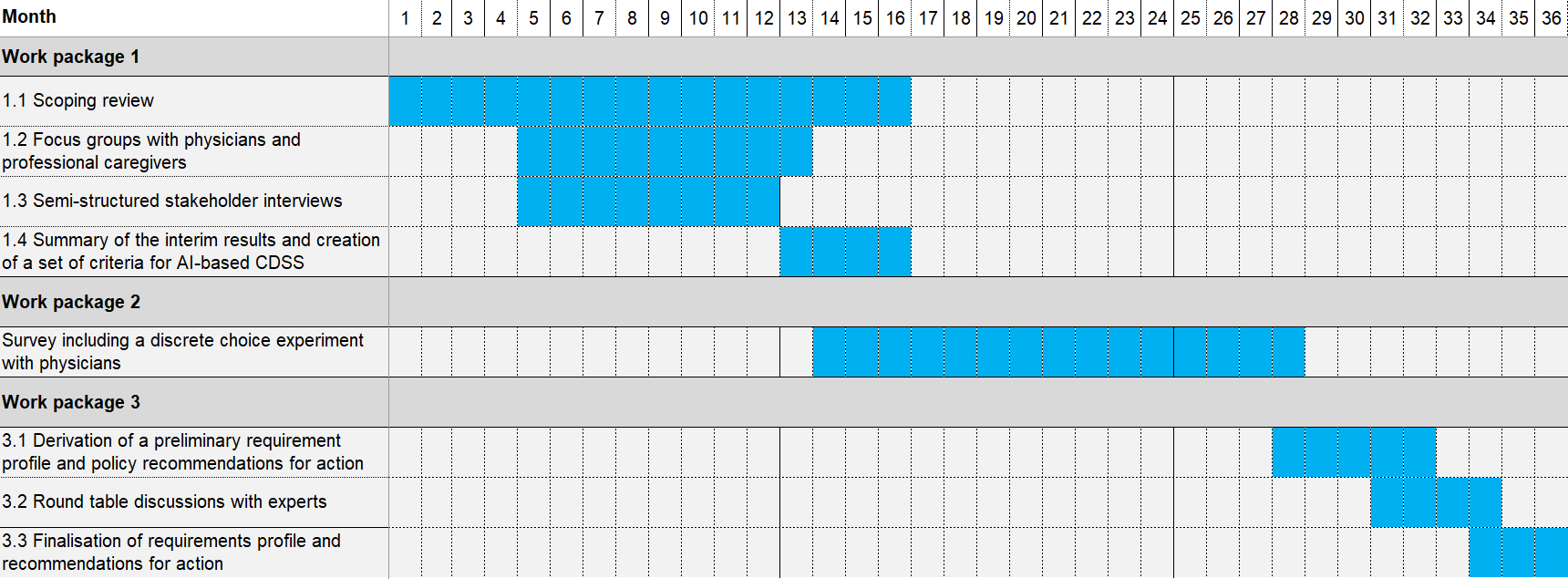

Supplement: Multimedia Appendix 1 [file resprot_v14i1e62704_app1.png]
